# Hidden in Plain Sight: Peritoneal Tuberculosis Mimicking Ovarian Neoplasm—A Case Report

**DOI:** 10.3390/tropicalmed10120346

**Published:** 2025-12-09

**Authors:** Dolfus Santiago Romero-Rojas, Andrés Rojas-Torres, Brenda Amell-Barón, David Serna, Luis Vasquez-Pinto, Luis Eduardo Barrera-Herrera, Javier Iván Lasso-Apraez

**Affiliations:** 1 Pulmology Department, Hospital Universitario San Ignacio, Bogotá 110231, Colombia; anrojas@husi.org.co (A.R.-T.); bramell@husi.org.co (B.A.-B.); dserna@husi.org.co (D.S.); luvasquez@husi.org.co (L.V.-P.); jilasso@husi.org.co (J.I.L.-A.); 2 Pathology Department, Hospital Universitario San Ignacio, Bogotá 110231, Colombia; lebarrera@husi.org.co

**Keywords:** peritoneal tuberculosis, disseminated tuberculosis, peritoneal carcinomatosis

## Abstract

Tuberculosis remains the deadliest infectious disease worldwide. Among extrapulmonary forms, peritoneal tuberculosis stands out as a rare and challenging diagnosis, often mistaken for intra-abdominal neoplasms or peritoneal carcinomatosis. The clinical, paraclinical, and imaging findings are similar and sometimes indistinguishable between the two entities, making the diagnosis a challenge for the treating physician. Here, we present the case of a young woman with chronic constitutional symptoms who presented to the emergency department with abdominal pain and ascites. An initial differential diagnosis of peritoneal carcinomatosis was considered based on findings in the peritoneal fluid and abdominal CT scan, leading to diagnostic laparoscopy. Histopathological examination of the samples revealed non-caseating granulomas involving the peritoneum, with no findings suggestive of malignancy. Subsequently, molecular testing for *Mycobacterium tuberculosis* was positive in the biopsies and peritoneal fluid, establishing the diagnosis of peritoneal tuberculosis. This case highlights the importance of awareness of peritoneal tuberculosis as a differential diagnosis of ascites and its significant potential to mimic other pathologies.

## 1. Introduction

Tuberculosis (TB) is one of the oldest known diseases in human history and remains the most lethal infectious disease worldwide today. Recent estimates indicate an annual incidence of 134 cases per 100,000 inhabitants, causing approximately 1.25 million deaths each year, despite the availability of effective treatment for the infection [1,2]. Globally, TB prevalence is higher in tropical countries, with greater incidences in South Asia, sub-Saharan Africa, and Latin America, primarily affecting migrants, prisoners, refugees, healthcare workers, and individuals living with HIV [2]. The main presentation of the disease is pulmonary TB, accounting for about 80% of diagnoses; however, *Mycobacterium tuberculosis* can infect various systems, causing extrapulmonary TB, defined as clinical or bacteriological diagnosis of infection in any organ other than the lungs [3,4]. It can involve the pleura, central nervous system, kidneys, ovaries, and peritoneum, among others [3,4].

Peritoneal TB accounts for only about 5% of extrapulmonary TB cases but poses a significant diagnostic challenge for clinicians. It can mimic other conditions such as peritoneal carcinomatosis or even primary abdominal or gynecological tumors, sometimes requiring invasive interventions like laparotomy or laparoscopy for definitive diagnosis [5,6]. Its symptoms are nonspecific, characterized by insidious onset over months, including fever, weight loss, progressive abdominal pain, and ascites [6,7,8]. Key tomographic features of peritoneal TB include diffuse and uniform thickening of the peritoneum with nodularity, mesenteric or para-aortic lymphadenopathies with hypodense centers, hepatosplenomegaly, omental thickening, and ascites [9,10]. These findings, in the absence of other clinical context, may be mistaken for neoplastic pathology with peritoneal extension [10,11], delaying diagnosis and treatment.

Therefore, we present the case of a woman from Cali, Colombia, with long-standing constitutional symptoms, initially suspected of having advanced ovarian cancer based on tomography findings, with an unexpected final diagnosis of peritoneal tuberculosis following further studies.

## 2. Case Presentation

**Figure 1:**
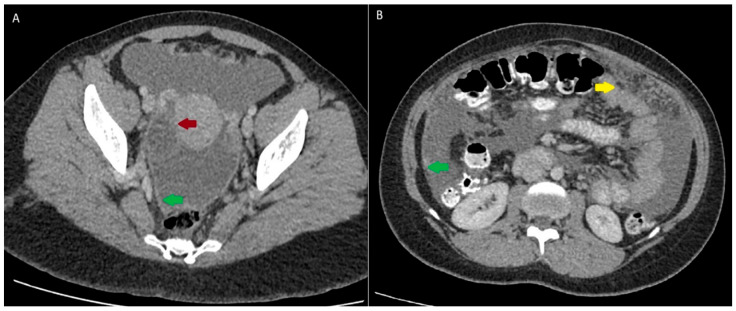
Abdominopelvic computed tomography. (**A**) Thin-walled hypodense right adnexal lesion (red arrow) and smooth thickening of the peritoneum (green arrow). (**B**) Nodular thickening of the peritoneum (green arrow) and nodular thickening of the greater omentum (yellow arrow).

The patient was a 23-year-old female nursing student from Cali, Colombia, with no previous remarkable medical background. The patient resided in an urban area and had no known exposure to infection from patients with respiratory symptoms. She presented with a six-month history of diffuse abdominal pain, asthenia, unquantified weight loss, and abdominal distension, leading to hospitalization for further evaluation. Ascites was documented, and paracentesis was performed. Analysis of peritoneal fluid revealed a low serum–ascites albumin gradient (SAAG) of 0.9 g/dL, predominantly lymphocytic cells, and negative microbiological tests, including a negative TB molecular test. An abdominal CT scan showed abundant ascites, inguinal lymphadenopathies, and nodular thickening of the peritoneum and omentum suggestive of peritoneal carcinomatosis, possibly of gynecological origin due to a right adnexal cystic lesion (Figure 1).

Additionally, elevated CA-125 tumor marker (424 U/mL) was documented, and based on these findings, the initial differential diagnosis was peritoneal carcinomatosis.

A diagnostic laparoscopy was performed, involving resection of the lesion on the omentum, drainage of ascitic fluid, and biopsy of the identified peritoneal lesions. Intraoperative findings were consistent with peritoneal carcinomatosis, reporting “omental cake,” involvement of the diaphragmatic domes and the round ligament of the liver, retraction of the root of the mesentery with involvement of the uterine serosa and fallopian tubes, and 500 mL of ascitic fluid. Notably, the ovaries had a normal appearance and regular microbiological tests were negative.

Fever and abdominal pain worsened, and severe symptomatic anemia with hemodynamic deterioration raised the suspicion of a surgical complication of the previous procedure and prompted urgent exploratory laparotomy. This time, there was a thickened peritoneum with multiple whitish nodular lesions, along with a 300 mL hemopurulent abdominal collection. Given the acute presentation with fever and systemic inflammatory response, TB tests on peritoneal fluid and tissue biopsies were performed. The analysis of the peritoneal fluid showed elevated leukocytes with neutrophilic predominance, glucose level < 5 mg/dL, and protein of 2.69 g/dL, suggestive of secondary peritonitis, and antibiotics were initiated.

**Figure 2:**
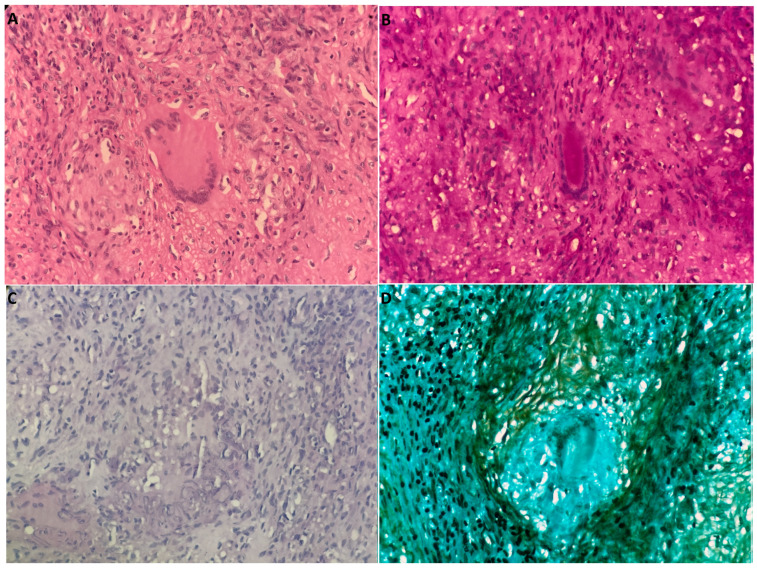
Histopathological examination of peritoneal tissue biopsies. (**A**) Granulomatous inflammation with multinucleated giant cells (H&E). (**B**–**D**) Special histochemical stains including Ziehl–Neelsen, PAS, and Gomori methenamine silver were performed at 4000× magnification and yielded negative results for fungal elements and other microorganisms.

Molecular testing on these new samples ultimately reported a positive polymerase chain reaction (PCR) test for *Mycobacterium tuberculosis* in both the peritoneal fluid and the biopsied tissue. Histopathological examination revealed, in hematoxylin and eosin-stained sections, multiple well-formed granulomas composed of epithelioid histiocytes with surrounding lymphocytic infiltrates (Figure 2).

The patient was subsequently evaluated by the pulmonology team, and anti-tuberculous treatment was prescribed. Further tests ruled out active pulmonary tuberculosis, and the HIV test was negative. The patient’s symptoms improved, and she was discharged in stable condition with a favorable follow-up in an outpatient care clinic.

## 3. Discussion

In 2023, tuberculosis was the leading infectious cause of death worldwide, with approximately 1.3 million cases, surpassing SARS-CoV-2 infection and HIV/AIDS [12]. Around 7.5 million cases were reported to the WHO; however, it is estimated that 10.6 million people were affected globally [12]. Pulmonary TB is the most common form of the disease, accounting for 80 to 85% of cases; the remaining percentage is classified as extrapulmonary TB. Within this classification, peritoneal TB accounts for 2 to 3% of all tuberculosis cases [13]. Peritoneal TB is a complex and challenging diagnosis due to its insidious onset, nonspecific symptoms, and low prevalence. Therefore, it should be considered among differential diagnoses when findings compatible with peritoneal carcinomatosis are observed [14], which was the initial diagnosis in our patient.

**Figure 3:**
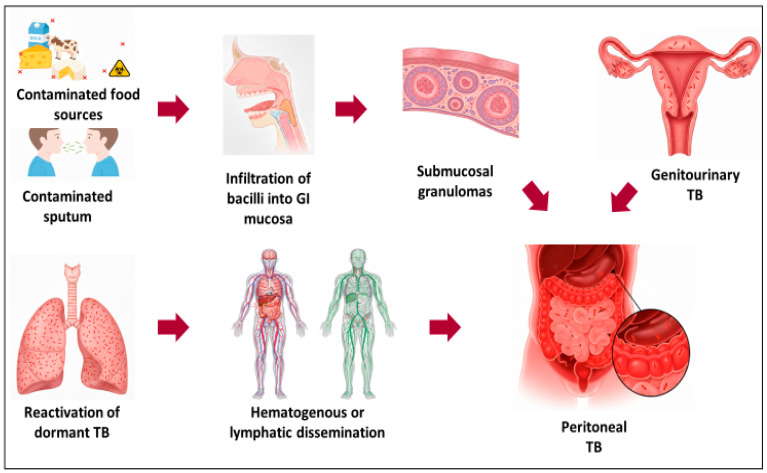
Diagram illustrating the routes of *Mycobacterium tuberculosis* dissemination to the peritoneum, adapted from Putra et al. [14].

Mycobacteria can reach the peritoneum through various routes, including a hematogenous pathway, ingestion of sputum (in cases of pulmonary TB), contaminated food (especially for *M. bovis*), or direct extension from an adjacent focus [13]. The figure adapted from the case series by Putra et al. [14] visually describes these pathways leading to the development of peritoneal tuberculosis (Figure 3).

When infected with the bacillus, the peritoneum exhibits hypervascularization, thickening granuloma formation with subsequent nodularity, and increased fluid production due to the inflammatory environment, resulting in ascites.

The symptoms of this condition develop insidiously; most patients present with abdominal pain (50–100%), distension secondary to ascites (40–73%), and weight loss (14–18%). Fever is variable, ranging from 20% to 60% depending on the case series reviewed [15,16,17,18]. Our patient initially presented with abdominal pain and ascites, associated with nocturnal sweating, unquantified weight loss, and a sensation of gastric fullness; however, she did not experience fever during her disease course.

Regarding laboratory findings, the ascitic fluid typically shows high protein levels (>2.5 g/dL) with a serum–ascites albumin gradient (SAAG) less than 1.1 g/dL, as seen in this patient [15]. Elevated adenosine deaminase (ADA) levels in the fluid (>30 U/L) are highly suggestive of tuberculosis [15]. Unfortunately, this test was not available during the care of this patient. Nevertheless, it is important to note that despite the high sensitivity and specificity of ADA for TB diagnosis, it can also be elevated in cases of peritoneal malignancy and purulent or hemorrhagic ascites, and may be falsely low in immunodeficient patients, liver cirrhosis, or early stages of peritoneal TB [15]. In this case, elevated CA-125 levels initially supported the diagnosis of secondary peritoneal carcinomatosis due to ovarian cancer. However, this biomarker can be elevated in other conditions causing abdominal and pelvic inflammation such as endometriosis, hepatitis, pancreatitis, and peritonitis [15,16]. In the series by Koc and colleagues, elevated CA-125 levels were documented in patients initially suspected of ovarian cancer who were ultimately diagnosed with peritoneal TB, although not all patients showed elevated levels [16]. It is important for clinicians to consider the multiple causes of tumor marker elevations and take into account the underlying pathophysiology behind these increases.

Regarding molecular tests, the GeneXpert MTB/RIF assay was endorsed by the WHO in 2010 following publication in the *New England Journal of Medicine* evaluating its performance [17]. Subsequently, other PCR tests were developed. Hodille et al. evaluated its performance in pulmonary and extrapulmonary samples, reporting a moderate sensitivity of 80% (95% CI, 65.4–90.4) [18]. In contrast, Bisognin et al. documented a sensitivity of 86.3% (95% CI, 76.7–92.9) and a specificity of 100% (95% CI, 95.5–100), analyzing urine, tissue biopsies, cavity fluid, and gastric aspirates [19]. In our patient, the first PCR test performed was negative, probably due to the low bacillary load and the sensitivity mentioned previously.

Imaging is a fundamental tool for guiding diagnosis. In peritoneal TB, a computed tomography (CT) scan of the abdomen typically reveals ascites and findings consistent with carcinomatosis [14,15]. Sohail et al. documented CT features in patients with peritoneal carcinomatosis and TB, highlighting potential approaches to differentiate between them, establishing that macro- and micronodules are more common in peritoneal TB than in carcinomatosis, as well as smooth infiltration of the omentum and splenomegaly [10]. Ramanan and colleagues described a new sign to differentiate carcinomatosis from peritoneal tuberculosis. The “omental rim” is defined as a uniform, enhanced border that partially or completely outlines the omentum on the venous phase of CT. This sign showed a sensitivity of 96% for the diagnosis of peritoneal TB [20]; however, this sign was not evident in our patient.

Finally, intraoperative findings suggestive of peritoneal TB include avascular membranous adhesions, peritoneal inflammation, whitish or yellowish tuberculomas, a thickened peritoneum, and ascites [16,17].

Treatment for peritoneal tuberculosis remains generally the same as that for pulmonary TB, and ascites should resolve in the first three months of therapy; however, the second phase of the regime may be extended to seven months if needed. Surgical intervention is reserved for complex cases with fibrotic and extensive granulation tissue disease. Steroids are not routinely recommended due to lack of evidence supporting their use [21,22].

## 4. Conclusions

Peritoneal tuberculosis is a rare form of tuberculosis. Its diagnosis should be based on the patient’s origin, risk factors (such as cirrhosis and HIV), clinical presentation, imaging and surgical findings, and microbiological identification. Sometimes, the clinical features of this disease can be nonspecific, prompting surgical interventions such as exploratory laparoscopy for definitive diagnosis. It is important to note that the initial PCR test on our patient’s peritoneal fluid was negative; therefore, if clinical suspicion persists, additional diagnostic tests should be performed. Moreover, peritoneal carcinomatosis can mimic tuberculosis infection, making multidisciplinary assessment, high clinical suspicion, and appropriate paraclinical studies essential to reach the correct diagnosis.

## Data Availability

The original contributions presented in this study are included in the article. Further inquiries can be directed to the corresponding author.
